# Serological report of influenza a (H7N9) infections among pigs in Southern China

**DOI:** 10.1186/s12917-014-0203-x

**Published:** 2014-09-02

**Authors:** Pei Zhou, Malin Hong, Mary M Merrill, Huamei He, Lingshuang Sun, Guihong Zhang

**Affiliations:** 1 Key Laboratory of Animal Disease Control and Prevention of the Ministry of Agriculture, College of Veterinary Medicine, South China Agricultural University, 483 Wushan Road, Tianhe District, Guangzhou 510642, China; 2 Department of Environmental & Global Health, College of Public Health & Health Professions, University of Florida, 101 S. Newell Dr, Gainesville 32610, FL, USA

**Keywords:** H7N9, AIV, Pig

## Abstract

**Background:**

In 2013, a novel H7N9 avian influenza virus (AIV) was isolated from ill humans in Shanghai and Anhui Province, China. Since then, the virus has spread quickly throughout China. Previous isolation of H7N2 virus from swine suggests that additional H7 subtype AIVs may be transmitted through pigs. However, prior to the recent zoonosis of H7N9, there were very few studies on the seroprevalence of the H7 subtypes in this species. Thus, there is a need to perform serological surveys for novel H7N9 as well as other H7 subtype AIVs in swine. This surveillance may help us understand risk factors for outbreaks of influenza A (H7N9) virus.

**Results:**

Only 2.0% (26/1310) of the pig sera had antibodies with an HI titer ≥1:20, and none had an MN titer ≥1:80, against the H7 antigen. Thus, no samples were found to be positive against H7N9. However, 13.6% (178/1310) of the pig sera had antibodies with HI titer ≥1:20 and 8.5% (112/1310) by MN titer ≥1:80 against H9 antigen. Thirty-seven percent (484/1310) of the pig sera had antibodies with HI titer ≥1:20 and 18.2% (238/1310) had MN titer ≥1:80 against pandemic 2009.

**Conclusions:**

Pigs in southern China have been shown to be infected with multiple avian influenza viruses. As the prevalence of novel influenza A viruses (e.g., H7N9 avian influenza virus) may be increasing among poultry in China, similar seroepidemiological studies of pigs should be conducted in the future.

## Background

Among the 17 hemagglutinin (HA) subtype viruses detected among domestic birds, wild birds and bats, H7 subtypes have occasionally infected humans and generated significant public health concerns. In 2013, a novel H7N9 avian influenza virus (AIV) was isolated from ill humans in Shanghai and Anhui Province, China [1].Since then, the virus has spread quickly throughout the country. As of August 11, 2013, a total of 134 human H7N9 infections (45 deaths) in China had been reported to the World Health Organization (WHO) [2]. Recent research has shown that the novel H7N9 AIV may be transmissible between mammals (ferrets) [3],[4]. The H7N9 AIV has also been isolated from one retail pork worker in Shanghai. H7N9 zoonosis was not preceded by overt epizootics in domestic poultry or other avian species in the wild, and the source of human infection remains to be definitively established [5]. In response to the reported human infections with H7N9 virus, the Ministry of Agriculture of the People’s Republic of China expanded and enhanced surveillance in live bird markets and poultry farms, as well as in swine farms and slaughterhouses, across the whole country, particularly in the affected region and surrounding provinces of southern China. Within six weeks of the initial case report, testing of tens of thousands of samples from poultry and their environment resulted in the identification of 51 H7N9 virus isolates from the provinces of Anhui, Guangdong, Zhejiang, Fujian, and Jiangsu, as well as the Shanghai municipality, mostly from live poultry markets [6]. For multiple reasons, it seems biologically plausible that pigs could be involved in the ecology of this emergent H7N9 virus in southern China. Some large-scale swine farms are adjacent to lakes that are home to multiple species of wild birds. In southern China, many farms raise pigs and poultry in close proximity. Recently, avian-origin H7N2 influenza viruses have been isolated from pigs in South Korea [7]. The isolation of swine H7N2 virus suggests zoonotic significance, highlighting the probable transmission of H7 subtype AIVs through pigs and prompting us to perform surveillance in this species. Prior to the recent zoonosis of H7N9, there were very few studies on the seroprevalence of the H7 subtypes in pigs. Serologic surveys are urgently needed to help us understand outbreaks of influenza A (H7N9) virus. Thus, we conducted surveillance from January 2011 to November 2012 in the Jiangsu, Zhejiang, Guangdong and Fujian Provinces in eastern China to evaluate whether pigs were infected with the novel H7N9 virus.

## Methods

Swine farms in southern China vary in size from large-scale farms (>3000 pigs raised per year) to backyard farms (<100 pigs raised per year). Farms often raise pigs indoors, have little biosafety and seldom use swine influenza vaccines. Many pigs on swine farms have contact with wild birds or domestic poultry. We worked with local veterinary department leaders who regularly inspect swine farms in Jiangsu, Zhejiang, Guangdong and Fujian Provinces of southern China, where novel H7N9 AIV are currently circulating or were frequently detected in 2013. For inclusion in the study, we selected swine farms by location (to generate a geographically balanced sample of farms), and we studied only pigs on large-scale farms that denied having a swine influenza vaccine program. Pigs were selected using a stratified random sampling method. Three groups of pigs were selected from each farm: weaning pigs, finishing pigs, and sows. No anesthesia was used during phlebotomy. All the owners of the swine farms gave permission for their animals to be sampled in this study. Pigs were restrained by rope or wire loop while a health professional performed the 4.0-5.0 mL serum draw. Our sampling processes were assisted by local authorities and licensed veterinarians. The animal research in this study protocol was reviewed and approved by the Guangdong Center for Disease Control and Prevention.

As reported in previous studies [8], we employed a hemagglutination inhibition (HI) assay according to the WHO’s protocol. An H7 antigen (A/Guangdong/GH074/2013 (H7N9)) derived from the emergent H7N9 AIV was obtained from the College of Veterinary Medicine, South China Agricultural University. Negative control serum (pig serum) and positive control (mice serum) were also prepared by the College of Veterinary Medicine, South China Agricultural University. All serum samples were treated with a receptor-destroying enzyme [RDE, prepared by China National Influenza Center (CNIC)] and absorbed with erythrocytes to remove nonspecific inhibitors before use in the assays. Briefly, two-fold serial dilutions of serum samples were added to V-shaped micro titer plates, and 4 HA units of virus were added to each well. The mixture was incubated at room temperature for 35 minutes. Then, 1% (v/v) horse erythrocytes were added in each well. The plates were left at room temperature for 40 minutes. The HI titers were expressed as the highest dilution of serum producing complete inhibition of agglutination. All positive control serum specimens had HI titers of 1:20 or more. We isolated the other AIV antigens used in this study: low pathogenic avian influenza viruses (LPAIV) A/chicken/Guangdong/V/2008 (H9N2), at the College of Veterinary Medicine, South China Agricultural University. The LPAIV A/chicken/Guangdong/V/2008 (H9N2), Beijing/1/94-like, was selected for a control as it is the most prevalent subtype of influenza virus in poultry in China [9]. The antigens A/California/7/2009pandemic (H1N1) 2009 virus, recently circulating among pigs in China, was also used for HI testing. Negative control serum and positive control serum specimens from different species were included in each plate to provide a full range of controls. Sera from pigs that were HI titer ≥ 20 by horse-RBC HI assay were confirmed with a microneutralization assay (MN) procedure recommended by the WHO [10]. Both the HI titer ≥ 1:20 and MN titer ≥ 1:80 were considered as positive evidence of previous H7N9 infection.

## Results

During the period between January 2011 and November 2012, we drew blood from a total of 1310 pigs from 120 farms in Jiangsu, Zhejiang, Guangdong and Fujian Provinces of southern China. These 4 provinces have had numerous recent detections of H7N9. A total of 172 of the 1310 pigs had clinical signs of influenza-like infection (cough, sneezing or heavy nasal mucus). The results from the 1310 pig blood samples were obtained by HI assay and MN assay. HI titers of ≥1:20 were detected in 178 of the 1310 serum samples using the H9N2 AIV antigens. Only 26 of the 1310 samples had HI titers of ≥1:20 (2/26 with HI titer = 1:40) using the H7N9 AIV antigens. Although 112 of the 178 H9N2 AIV HI-positive samples were also positive by the MN assay (MN titer ≥1:80), none of the 26 H7N9 AIV HI-positive samples were positive by the MN assay (MN titer ≥1:80), revealing that no samples were positive for H7N9. As a control, 37.0% (484/1310) of sera were found positive by HI assay (HI titer ≥1:20) for H1N1 pdm09, and 18.2%(238/484)of these HI-positive samples were also positive by the MN assay (MN titer ≥1:80) (Table 1).

**Table 1:** Characteristics an influenza serological assay results of pigs sampled with serum specimen collections, January 2011 to November 2012

**Location**	**Pigs studied**	**Number sampled**	**Number (%) with ILI**	**Number (%) with serological evidence of avian H9N2 infection**		**Number (%) with serological evidence of avian H7N9 infection**		**Number (%) with serological evidence of H1N1 pdm09 infection**	
				**HI assay ≥1:20**	**MN assay ≥1:80**	**HI assay ≥1:20**	**MN assay ≥1:80**	**HI assay ≥1:20**	**MN assay ≥1:80**
Jiangsu	Weaning pigs	84	18(21.4)	8(9.5)	4(4.8)	0(0)	0(0)	22(26.2)	14(16.7)
	Finishing pigs	120	12(10)	14(11.7)	8(6.7)	2(1.7)	0(0)	48(40.0)	22(18.3)
	Sows	92	12(13.0)	18(19.5)	12(13.0)	4(4.3)	0(0)	28(30.4)	18(19.6)
Zhejiang	Weaning pigs	92	10(10.9)	6(6.5)	2(2.2)	0(0)	0(0)	34(37.0)	10(10.9)
	Finishing pigs	124	20(16.1)	14(11.3)	8(6.5)	0(0)	0(0)	40(32.3)	26(21.0)
	Sows	108	18(16.7)	24(22.2)	16(14.8)	4(3.7)	0(0)	40(37.0)	20(18.5)
Guangdong	Weaning pigs	152	22(14.5)	10(6.6)	4(2.6)	0(0)	0(0)	50(32.9)	16(10.5)
	Finishing pigs	124	8(6.5)	16(12.9)	12(9.7)	2(1.6)	0(0)	36(45.2)	28(22.6)
	Sows	152	24(15.8)	32(21.1)	26(17.1)	8(5.3)	0(0)	66(43.4)	36(23.7)
Fujian	Weaning pigs	78	14(17.8)	6(7.7)	4(5.1)	0(0)	0(0)	26(33.3)	10(12.8)
	Finishing pigs	94	6(6.4)	12(12.8)	6(6.4)	4(4.3)	0(0)	36(38.3)	18(19.1)
	Sows	90	8(8.9)	18(20.0)	10(11.1)	2(2.2)	0(0)	38(42.2)	20(22.2)
Total		1310	172(13.3)	178(13.6)	112 (8.5)	26(2.0)	0(0)	464(35.4)	238(18.2)

ILI = influenza-like-illness; HI = horse RBC hemagglutination inhibition assay; MN = microneutralization assay; H9N2 = A/chicken/Guangdong/V/2008(H9N2); H1N1 = A/California/7/2009pandemic (H1N1) ; H7N9 = A/Guangdong/GH074/2013(H7N9).

**Figure 1:**
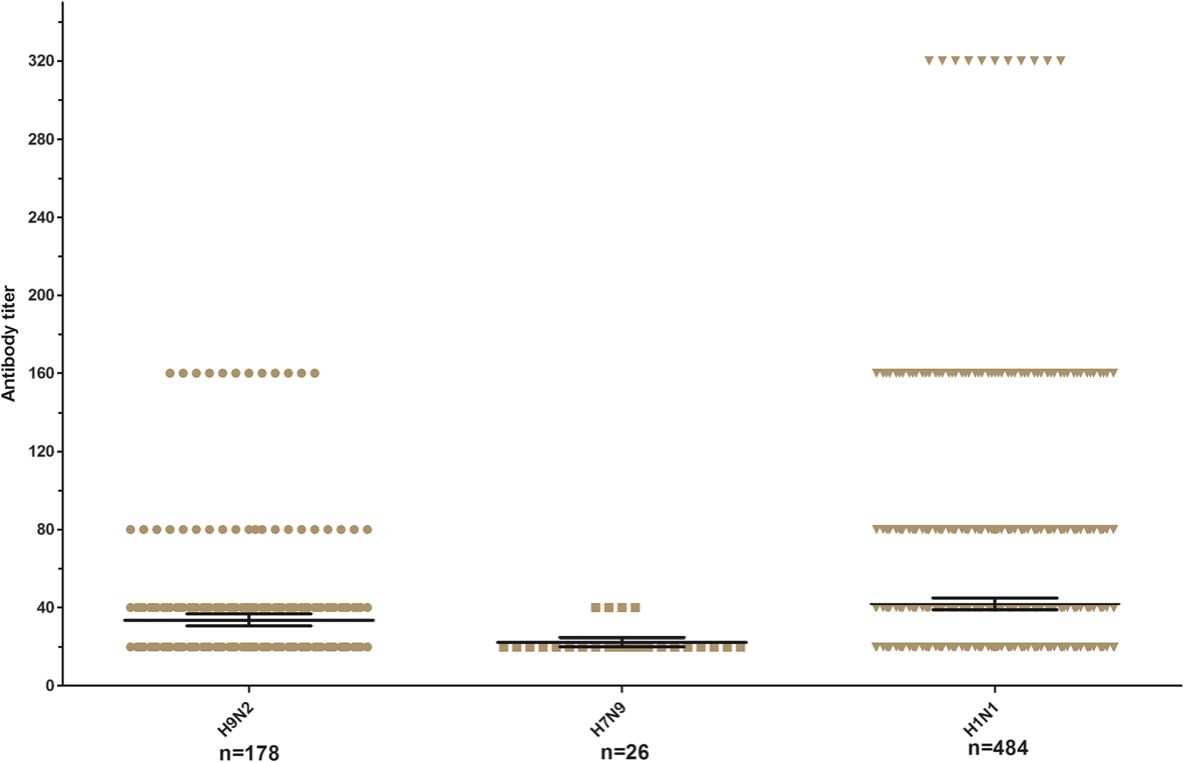
**The geometric mean antibody titers of H9N2 AIV, H7N9 AIV and H1N1 pdm09 infections from January 2011 to November 2012 using a hemagglutination inhibition (HI) assay.** Geometric mean titers and 95% CIs of antibody titers against subtype H9N2, H7N9 and H1N1 are indicated by long and black short horizontal lines. Samples titers indicated by borrow symbols.

None of the four provinces studied had statistically significant different rates of swine infection with avian influenza virus. However, by HI assay, samples from 2012 showed an increased risk of elevated antibodies against avian influenza virus H7N9 as compared to samples from 2011. Sows had a significant increased risk of elevated antibodies against avian influenza virus H7N9, and finishing pigs had increased risk (though not significant), compared to weaning pigs. Also, pigs without signs of influenza-like-illness had a significant increased risk of elevated antibodies to avian H9N2 and pdm09 H1N1 as compared to pigs with signs of influenza-like-illness. Influenza-like-illness had no significant effect on antibodies against H7N9 virus (Table 2). HI geometric mean antibody titers (GMT) of AIV H9N2 and H1N1 pdm09 were 33.44 and 40.98 respectively (Figure 1). The GMT of H7N9 was 22.25, indicating again that all serum samples were negative.

**Table 2:** Risk factors for elevated antibody against influenza A viruses by horse RBC hemagglutination inhibition assay (titer ≥1:20) among pigs sampled in Jiangsu , Zhejiang , Guangdong and Fujian Provinces China

	**A/chicken/Guangdong/V/2008(H9N2)**		**A/Guangdong/GH074/2013(H7N9)**		**A/California/7/2009pandemic (H1N1)**	
**Risk factor**	**Number (%)**	**OR (95% CI)**	**Number (%)**	**OR (95% CI)**	**Number (%)**	**OR (95% CI)**
Location						
Jiangsu	40(13.5)	1.0[0.63-1.58]	6(2.0)	1.7[0.46-5.93]	98(33.1)	0.92[0.65-1.27]
Fujian	36(13.7)	1.0[0.63-1.63]	6(2.3)	1.9[0.52-6.72]	100(38.2)	1.1[0.81-1.59]
Guangdong	58(13.6)	1.0[0.65-1.52]	10(2.3)	1.9[0.59-6.16]	152 (29.2)	1.0[0.75-1.37]
Zhejiang	44(13.6)	reference	4(1.2)	reference	114(35.2)	reference
Pigs studied						
Sows	92(20.8)	3.3[2.13-5.10]	18(4.1)	35.4[2.13-590.30]	172(38.9)	1.3[0.10-1.75]
Finishing pigs	56(12.1)	1.7[1.09-2.75]	8(1.7)	15.2[0.84-264.4]	160(34.6)	1.0[0.83-1.46]
Weaning pigs	30(7.4)	reference	0(0.0)	reference	132(32.5)	reference
Time						
2012	103(17.7)	1.4[0.98-1.87]	20(3.4)	3.2[1.28-8.07]	252(43.3)	1.2[0.95-1.54]
2011	75(13.7)	reference	6(1.1)	reference	212(38.7)	reference
ILI						
No	170(14.9)	3.6[1.74-7.46]	24(2.1)	1.8[0.43-7.82]	390(34.3)	3.9[2.49-6.08]
Yes	8(4.6)	reference	2(1.2)	reference	24(14.0)	reference

Pigs were studied during the period January 2011 to November 2012.

## Discussion

China has been identified as a hot spot for the generation of novel influenza viruses. In this report, we identified large farms that seemed to have a high probability of exposure to the novel H7N9 virus and found no evidence of infection in pigs. The surveillance of pigs has focused on the subtype of H1, H3, H5 and H9 [11]-[13], while prior to the recent zoonosis of H7N9 [1],[3]-[5],[14],[15], there were very few studies on the seroprevalence of the H7 subtype. Our results matched the results reported previously, with an 8.5% prevalence of H9 infection. Although we found no definitive serological evidence of avian H7 influenza virus in swine in southern China, 2.0% of the specimens had an HI titer ≥1:20. This suggests that pig herds of southern China may have some potential risks of subtype H7 infection. While we agree that pigs have potential to serve as mixing vessels for the novel influenza virus generation [16],[17], this study found no evidence that pigs were involved in the ecology of the H7N9 virus.

Our study had a number of limitations. First, it is possible that results may have been confounded due to serologic cross-reactivity between different viral subtypes. In our study, the possibility of cross-reaction between subtypes was not evaluated. However, there were no positive serum against H7N9 via two serological methods (HI and MN), which indicates that no cross-reaction occurred between H7N9 and other avian viruses of this study. Second, we did not study pigs from small farms or slaughterhouses, and thus our study population may not be representative of all pigs in these provinces.

## Conclusions

The unique environment on swine farms and live animal markets (LAMs) in southern China provides many opportunities for wild aquatic birds, domestic poultry, and pigs to come in close contact with one another, thereby increasing the likelihood of interspecies transmission and generation of novel influenza viruses through reassortment [11],[18]. As novel viruses may change rapidly, it seems prudent to continue surveillance for the H7N9 virus among non-poultry domestic animal species such as pigs and dogs [19],[20]. Zhu et al. reported that pigs were infected productively by human-isolated H7N9 influenza virus and shed virus for 6 days [15]. Xu and colleagues reported rapid adaptation of avian H7N9 virus in pigs; the avian H7N9 virus replicated to a high titer after only one passage [14]. P. Horby suggests that a hidden epidemic of H7N9 in other animals may be well under way and that it will provide opportunities for further adaptation of H7N9 to mammals and for re-assortment with human- or pig-adapted viruses if H7N9 is circulating in the large populations of pigs [21]. Therefore, such studies are crucial in understanding and reducing risk factors for human H7N9 infection in China.

### Ethical approval

This study protocol was reviewed and approved by the Institutional Review Board of South China Agricultural University.

## Competing interests

The authors declare that they have no competing interests.

## Authors’ contributions

PZ and GZ designed the experiments. MH, LW, HH carried out the tests. PZ, MMM and LS drafted the manuscript. All authors have read and approved the final manuscript.
